# Quality of Life and Safety Outcomes after First-Line Treatment of Symptomatic AF with Cryoablation or Drug Therapy: A Meta-Analysis of Randomized Controlled Trials

**DOI:** 10.31083/j.rcm2502071

**Published:** 2024-02-20

**Authors:** Qingchun Song, Haoyu Tan, Benli Yang, Hongduan Liu, Chengming Fan

**Affiliations:** ^1^Department of Cardiovascular Surgery, Second Xiangya Hospital, Central South University, 410008 Changsha, Hunan, China

**Keywords:** atrial fibrillation, cryoablation, anti-arrhythmic drug, meta-analysis, quality of life, first-line treatment

## Abstract

**Background:**

Cryoablation has emerged as a recognized interventional strategy for the treatment of atrial fibrillation (AF). Numerous trials have investigated cryoablation as a first-line therapy for AF. This meta-analysis aimed to evaluate the impact of cryoablation on quality of life (QoL) and safety outcomes compared to antiarrhythmic drugs (AADs) in patients with symptomatic AF.

**Methods:**

A comprehensive search of the PubMed, EMBASE, and Cochrane Library databases was conducted for randomized controlled trials (RCTs) comparing cryoablation and AADs as first-line treatments for AF until May 2023. Continuous outcome data were analyzed using mean differences (MDs) with 95% confidence intervals (CIs), and dichotomous outcome data were analyzed using relative risks (RRs) with 95% CIs. The primary outcomes assessed were QoL and serious adverse events.

**Results:**

Our analysis included four RCTs involving 928 patients. Cryoablation was associated with a significant improvement in the AF Effect on Quality of Life (AFEQT) score (3 trials; MD 7.46, 95% CI 2.50 to 12.42; *p* = 0.003; $I^{2}$ = 79%) and EQ-VAS score (2 trials; MD 1.49, 95% CI 1.13 to 1.86; *p* < 0.001; $I^{2}$ = 0%) compared to AAD therapy. Additionally, cryoablation demonstrated a modest increase in EQ-5D score from baseline compared to AAD therapy, with no statistically significance (2 trials; MD 0.03, 95% CI –0.01 to 0.07; *p* = 0.07; $I^{2}$ = 79%). Furthermore, the rate of serious adverse events was significantly lower with cryoablation compared to AAD therapy (4 trials; 11.8% vs. 16.3%; RR, 0.73; 95% CI, 0.54–1.00; *p* = 0.05; $I^{2}$ = 0%). Cryoablation was also associated with a reduction in overall adverse events, incidence of persistent AF, hospitalizations, and additional ablation. However, there was no significant difference in major adverse cardiovascular events and emergency department visits between the two treatment groups.

**Conclusions:**

Cryoablation, as a first-line treatment for symptomatic AF patients, significantly improved AF-specific quality of life and reduced serious adverse events, as well as overall adverse events, persistent AF, hospitalizations, and additional ablation compared to AADs.

## 1. Introduction 

Atrial fibrillation (AF), the most prevalent cardiac arrhythmia, affects approximately 37.6 million individuals worldwide [1]. Without preventive treatment, AF recurrence is likely to occur in 90% of patients [2]. Current guidelines recommend antiarrhythmic drugs (AADs) as the first-line treatment for maintaining sinus rhythm in symptomatic AF patients [3, 4]. However, the effectiveness of AAD therapy is somewhat limited, and a significant proportion of patients discontinue treatment due to severe side effects [5, 6]. In cases of AAD ineffectiveness or intolerance, catheter ablation is recommended and has been shown to be superior to additional AAD therapy [7, 8, 9].

Since 2005, several randomized clinical trials (RCTs) have compared radiofrequency ablation with AAD therapy in patients without prior AF ablation or AAD usage [10, 11, 12]. These trials have shown a modest reduction in AF recurrence but an increased occurrence of serious adverse events with ablation compared to AAD therapy [13]. Cryoballoon pulmonary vein isolation (PVI), first introduced in 2003, has emerged as another recognized method for ablating AF [14, 15]. Three similar RCTs have reported on the comparison of cryoablation and AAD therapy as first-line treatments for symptomatic AF [16, 17, 18]. Several meta-analyses of these studies have demonstrated improved outcomes in terms of AF recurrence and hospitalizations, with no significant difference in major adverse events [19, 20]. However, few reviews have focused on the quality of life (QoL) and safety outcomes associated with cryoablation as a first-line treatment. Therefore, the aim of this systematic review and meta-analysis is to further evaluate the impact of cryoablation as a first-line therapy compared to drug therapy on the QoL and safety of patients with symptomatic AF.

## 2. Methods 

### 2.1 Data Sources and Search Strategy

We conducted a comprehensive search of three databases, namely PubMed, Cochrane Library, and Web of Science, to retrieve relevant articles published up until May 1, 2023. Our search strategy did not impose any restrictions on language or year of publication. The literature was searched using the following words “random*” [tiab] AND ((“ablation” [tiab] OR “drug*” [tiab] OR “anti-arrhythmic” [tiab] OR “medica*” [tiab] OR “cryothermal” [tiab] OR “cryoablation” [tiab] OR “cryoballoon” [tiab] OR “cryotherapy” [tiab] OR “cryo*” [tiab]) AND (“AF” [tiab] OR “Atrial Fibrillation” [tiab] OR “Atrial Fibrillation” [MeSH Terms])).

### 2.2 Study Selection

In the present analysis, our objective was to include prospective RCTs that assessed the QoL and safety outcomes of cryoablation compared to AAD therapy as the first-line treatment for symptomatic AF patients. We applied the following inclusion criteria: (1) prospective RCTs with a minimum follow-up duration of 1 year, (2) studies comparing cryoablation with drug therapy as the initial treatment for symptomatic AF, and (3) availability of data on QoL and adverse events.

### 2.3 Data Extraction and Quality Appraisal

Two independent reviewers (QS and HT) screened the titles and abstracts of the retrieved articles and evaluated the full texts for eligibility. We extracted relevant information from each study using a structured data extraction form, including study characteristics (publication year, authors, follow-up duration, sample size, and study design), baseline characteristics (mean age, sex, prevalence of paroxysmal AF, hypertension, heart failure, prior stroke/transient ischemic attack-vascular disease, ${\mathrm{CHA}}_{2}$${\mathrm{DS}}_{2}$-VASc score, major comorbidity, and prior use of beta blockers and oral anticoagulation, and left ventricular ejection fraction (LVEF)), exposure details (procedural details of cryoablation and characteristics of AADs), and outcome measures (recurrence of any atrial arrhythmia, adverse events, hospitalizations, emergency department visits, crossover to alternate therapy, and additional ablation). The quality of included trials was assessed using the Cochrane risk of bias tool [21].

### 2.4 Clinical Outcomes

The primary outcomes of interest were improvements in the QoL and the occurrence of serious adverse events in AF patients following treatment. Secondary outcomes included overall adverse events, major adverse cardiovascular events (MACE), the incidence of persistent AF, hospitalizations, emergency department visits, and additional ablation in both treatment groups.

QoL was evaluated at baseline and the follow-up endpoint using the Atrial Fibrillation Effect on Quality of Life (AFEQT), European Quality of Life-5 Dimensions (EQ-5D), and visual analog scale (EQ-VAS) questionnaires. AFEQT is a disease-specific QoL questionnaire that provides an overall summary score, a treatment satisfaction score, and three domain scores encompassing symptoms, daily activities, and treatment concerns. EQ-5D is a general health-related QoL questionnaire consisting of a 5-question survey and a visual analog scale (EQ-VAS).

We compared the occurrence of serious adverse events, major adverse cardiovascular events, and overall adverse events as documented in the included trials. Serious adverse events were defined as events resulting in death, permanent impairment of a body structure or functional disability, interventions, or prolonged hospitalizations (>24 h), following the definitions provided in the respective studies. Major adverse cardiovascular events (MACE) included all-cause death, ischemic heart disease or acute coronary syndrome, unstable angina, heart failure, and stroke or transient ischemic attack (TIA) [22].

### 2.5 Statistical Analysis

A conventional meta-analysis was conducted to compare the outcomes of cryoablation and AAD therapy as initial treatments for symptomatic AF. We pooled the outcomes of studies with a follow-up assessment timing of more than 1 year for each trial. Continuous outcome data were presented as mean differences (MDs) with 95% confidence intervals (CIs), and dichotomous outcome data were presented as relative risks (RRs) with 95% CIs. The heterogeneity of the effect size across studies was tested using the Q statistic (*p* < 0.05 was considered heterogeneous) and the $I^{2}$ statistic ($I^{2}$ < 25% indicating low heterogeneity, 25% to 50% indicating moderate heterogeneity, or $I^{2}$ > 50% indicating high heterogeneity). In the presence of significant heterogeneity, a random-effects model was employed; otherwise, a fixed-effects model was used. Two-sided *p*-values < 0.05 were considered statistically significant.

All statistical analyses were performed using Review Manager 5.3 software (The Nordic Cochrane Center, Copenhagen, Denmark).

## 3. Results 

**Fig. 1.**
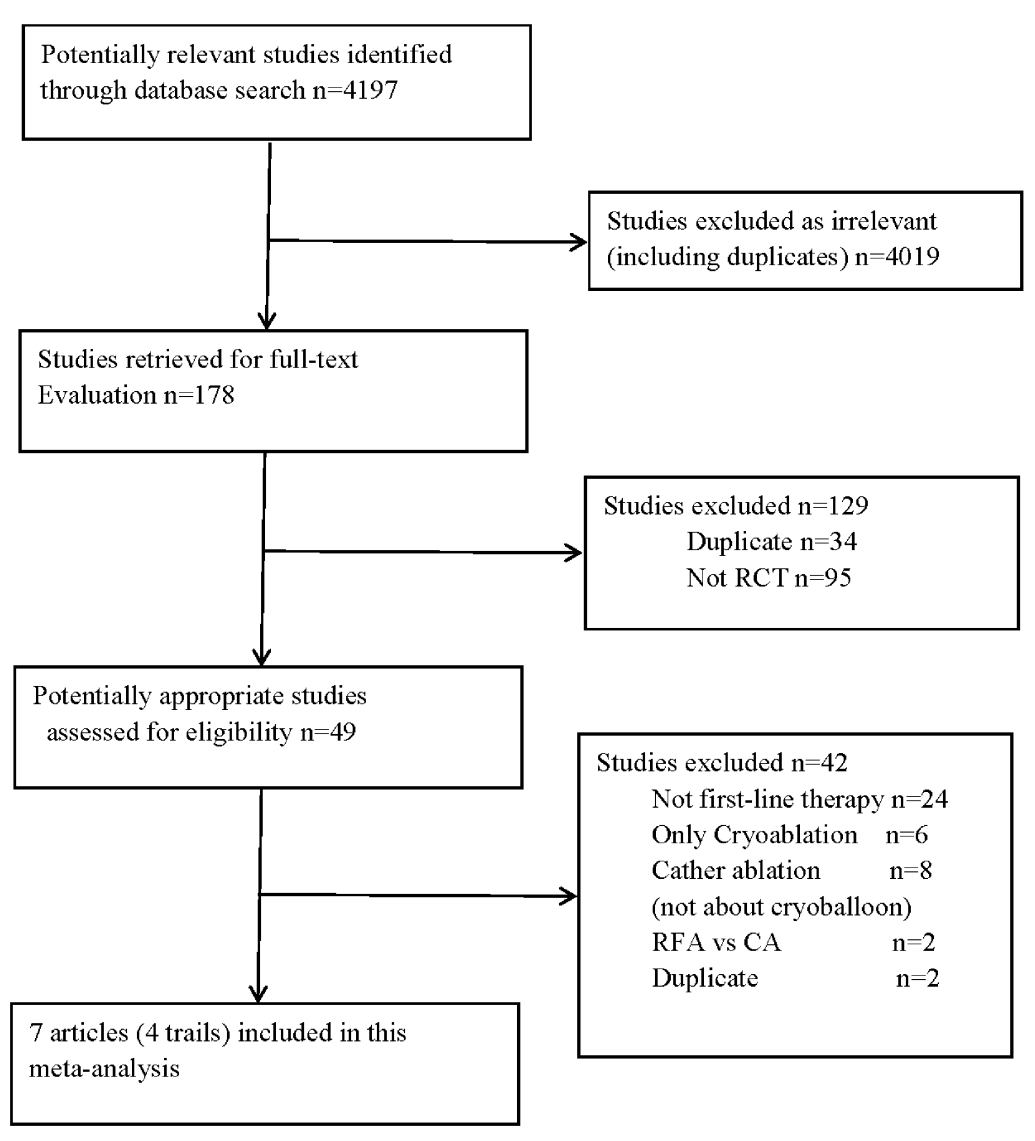
**Selection process of included studies**. RFA, radiofrequency ablation; CA, cryoablation; RCT, randomized controlled trial.

A total of 4,197 articles were identified in the literature search, of which 7 articles (4 trials) [16, 17, 18, 23, 24, 25, 26] met the inclusion criteria and were included in the analysis (Fig. 1).

### 3.1 Characteristics of the Eligible Trials 

All the included trials were prospective randomized trials that compared the efficacy and safety of cryoablation versus AAD therapy as the first-line treatment for symptomatic AF. The trials were open-label and utilized intention-to-treat analysis. Repeat ablation and crossover between the two treatment groups were permitted for patients who did not respond to initial therapy. Table 1 (Ref. [16, 17, 18, 24]) presents the main characteristics of the included studies.

**Table 1.** **Baseline characteristics of clinical trials**.

Variable (Ablation vs. AADs)	EARLY AF 2020 [16]	STOP AF 2020 [17]	CRYO-FIRST 2021 [18]	Ding *et al*., 2022 [24]
Age, Mean (SD), y	58.0 (12.0) vs. 60.0 (11.0)	60.0 (11.0) vs. 62.0 (11.0)	51.0 (13.0) vs. 54.0 (13.0)	60 (7.89) vs. 60.74 (10.16)
Male, No., %	112 (73.0) vs. 102 (69.0)	63 (61.0) vs. 57 (58.0)	76 (71.0) vs. 72 (65.0)	41 (40.20) vs. 42 (41.18)
Patients randomized, No.	154 vs. 149	104 vs. 99	107 vs. 111	102 vs. 102
Time since AF diagnosis, y	1.3 (2.2) vs. 1.7 (3.0)	1.3 (2.5) vs. 1.3 (2.3)	0.7 (1.5) vs. 0.8 (2.1)	2.75 (2.0) vs. 2.76 (2.0)
Paroxysmal AF, %	95.5 vs. 94.0	100.0 vs. 100.0	100.0 vs. 100.0	100.0 vs. 100.0
Total follow-up, y	3.0	1.0	1.0	3.0
Beta-blocker, No., (%)	85 (55.2) vs. 92 (61.7)	6 (6.0) vs. 9 (9.0)	54 (50.5) vs. 56 (50.5)	
Oral anticoagulation, No., (%)	67 vs. 64	69 vs. 69	36 vs. 44	
${\mathrm{CHA}}_{2}$${\mathrm{DS}}_{2}$-VASc, Mean (SD)	1.9 (1.0) vs. 1.9 (1.1)	NA	NA	1.65 ± 1.38 vs. 1.78 ± 1.30
Hypertension, No., (%)	57 (37.0) vs. 55 (36.9)	58 (56.0) vs. 57 (58.0)	33 (30.8) vs. 40 (36.0)	54 (52.94) vs. 47 (46.08)
Ischemic heart disease, No., (%)	12 (7.8) vs. 7 (4.7)	17 (16.0) vs. 14 (14.0)	4 (3.8) vs. 1 (0.9)	25 (24.51) vs. 27 (26.47)
Previous stroke or TIA, No., (%)	4 (2.6) vs. 5 (3.4)	2 (2.0) vs. 3 (3.0)	0 (0.0) vs. 1 (0.9)	8 (7.84) vs. 10 (9.80)
Stable heart failure, No., (%)	14 (9.1) vs. 14 (9.4)	1 (1.0) vs. 3 (3.0)	0 (0.0) vs. 0 (0.0)	7 (6.86) vs. 10 (9.80)
LAD, Mean (SD), mm	39.5 (5.0) vs. 38.1 (6.5)	38.7 (5.7) vs. 38.2 (5.4)	37.0 (5.9) vs. 38.0 (4.9)	38.29 (3.68) vs. 39.11 (3.89)
LVEF, Mean (SD), %	59.6 (7.0) vs. 59.8 (7.6)	60.9 (6.0) vs. 61.1 (5.9)	62.8 (5.4) vs. 63.7 (5.4)	60.91 (4.7) vs. 59.96 (5.0)

AADs, antiarrhythmic drugs; AF, atrial fibrillation; y, years; SD, standard deviation; vs., versus; ${\mathrm{CHA}}_{2}$${\mathrm{DS}}_{2}$-VASc, congestive heart failure, hypertension, age 75 years or older, diabetes, prior stroke/transient ischemic attack–vascular disease, age 65 to 74 years, sex category; TIA, transient ischemic attack; LVEF, left ventricular ejection fraction, LAD, Left atrial diameter; NA, not applicable.

928 patients were included in the 4 trials, with 467 randomized to cryoablation and 461 to drug therapy. The mean age of the participants was 58.42 years (SD: 11.86). The majority of participants (99%) had paroxysmal AF, and the average duration of AF was 1.54 years (SD: 2.39). Hypertension was the most common comorbidity. The mean LVEF was 60.95% (SD: 6.24), and the average diameter of the left atrium was 38.40 mm (SD: 5.31).

The risk of bias in each study was assessed using the Cochrane risk of bias tool, as shown in **Supplementary Fig. 1**. Allocation concealment was reported only in the EARLY AF trial, while the other three trials had an unclear risk of bias in this domain. Blinding of participants and study personnel was not feasible in any of the four studies. Outcome assessment was not reported in the STOP AF trial and Ding *et al*. [24]. The other domains were judged to have a low risk of bias.

Ablation procedures were performed using 23 mm or 28 mm second-generation cryoballoons (Arctic Front Advance, Medtronic) in all included studies. Pulmonary vein isolation (PVI) was achieved through a trans-septal puncture and an over-the-wire delivery technique. A minimum ablation duration of 3 minutes was recommended, with confirmation of PVI by entrance block and, where assessable, exit block. If PVI was not achieved, additional freeze applications with an alternative-sized cryoballoon or focal catheter (Freezor MAX, Medtronic) were allowed. The blanking period was 3 months. During the blanking period, antiarrhythmic drugs (excluding amiodarone) were permitted in the EARLY AF, STOP AF, and Ding *et al*. [24] trials. Use of AADs and repeat ablation were both allowed in the CRYO-FIRST trial. Commonly used AADs across all four trials included flecainide, propafenone, sotalol, amiodarone, and dronedarone. The follow-up duration of the studies ranged from 1 to 3 years (Table 2, Ref. [16, 17, 18, 24]).

**Table 2.** **Methods and outcome endpoints in the included randomized trials**.

	Inclusion criteria	Exclusion criteria	Ablation method	AAD therapy	AAD therapy after ablation	Primary endpoint	Secondary endpoint
EARLY AF, 2020 [16]	symptomatic AF and at least one episode of AF detected within 24 months	Regular use of a class I or III ADDs. Previous LA ablation or surgery. AF due to reversible cause. NYHA class III or IV congestive heart failure. LVEF ≤35% or hypertrophic cardiomyopathy	With 23- or 28-mm second-generation cryoballoon, using a trans-septal puncture and an over-the-wire delivery∖technique to undergo PVI. additional freeze applications were permitted if PVI not achieved	Flecainide Propafenone Sotalol Amiodarone Dronedarone	ADDs (excluding amiodarone) were allowed till five half-lives before the end of the blanking period	First recurrence of any atrial tachyarrhythmia lasting ≥30 seconds	First recurrence of symptomatic atrial arrhythmia, arrhythmia burden, QoL, success of multiple ablation procedures, health care utilization, serious adverse events.
	Age >18 years						
STOP AF, 2020 [17]	recurrent symptomatic PAF within 6 months	Treatment with class I or III ADDs. Prior persistent AF. LA diameter ≥5.0 cm. Prior LA ablation or surgical procedure. NYHA class III or IV congestive heart failure or known LVEF ≤45% Pacemaker or defibrillator implant		Flecainide Propafenone Sotalol Amiodarone Dronedarone	AADs (excluding amiodarone) was permitted for up to 80 days after the ablation	atrial arrhythmia recurrence for ≥30 seconds during ambulatory monitoring or for ≥10 seconds on a 12-lead ECG	QoL, health care utilization, serious adverse events, initial success of the procedure, procedural characteristics
	18 to 80 years of age						
CRYO-FIRST, 2021 [18]	recurrent symptomatic PAF who were drug naive structurally normal heart	AF due to reversible cause. Previous LA ablation. Previous cardiac surgery. Permanent pacemaker or defibrillator implant. Typical atrial flutter		Flecainide Propafenone Sotalol Amiodarone Dronedarone	AADs were allowed during the first 90 days after the index procedure	freedom from any AA recurrence lasting >30 s	SAEs and recurrence of patient-reported symptomatic palpitations
	18 to 75 years of age						
Ding *et al*., 2022 [24]	symptoms PAF and experienced ≥1 episodes of AF documented by Holter or 12-lead ECG within 6 months	previous LA ablation, acute coronary syndrome, LA size >50 mm, persistent AF, LVEF <40%, intracardiac thrombi, a reversible cause of AF, and decompensated heart failure.		Propafenone	amiodarone was excluded and other class I or III AADs were used for ≤8 weeks. Subjects were maintained on anticoagulation therapy for ≥3 months	first occurrence of persistent atrial tachyarrhythmia following a 90-day blanking period serious adverse events	event rates of the progression from paroxysmal AF to persistent atrial tachyarrhythmia at 1 and 2 years
	18–80 years old			Dronedarone			
	no regular drug use >2 weeks			Sotalol			
				Amiodarone			

ADDs, antiarrhythmic drugs; LA, left atrial; AF, atrial fibrillation; NYHA, New York heart association congestive; PAF, proximal atrial fibrillation; LVEF, left ventricular ejection fraction; ECG, electrocardiogram; SAEs, serious adverse event; QoL, quality of life; PVI, pulmonary vein isolation; AA, atrial arrhythmia.

### 3.2 Primary Outcome

**Fig. 2.**
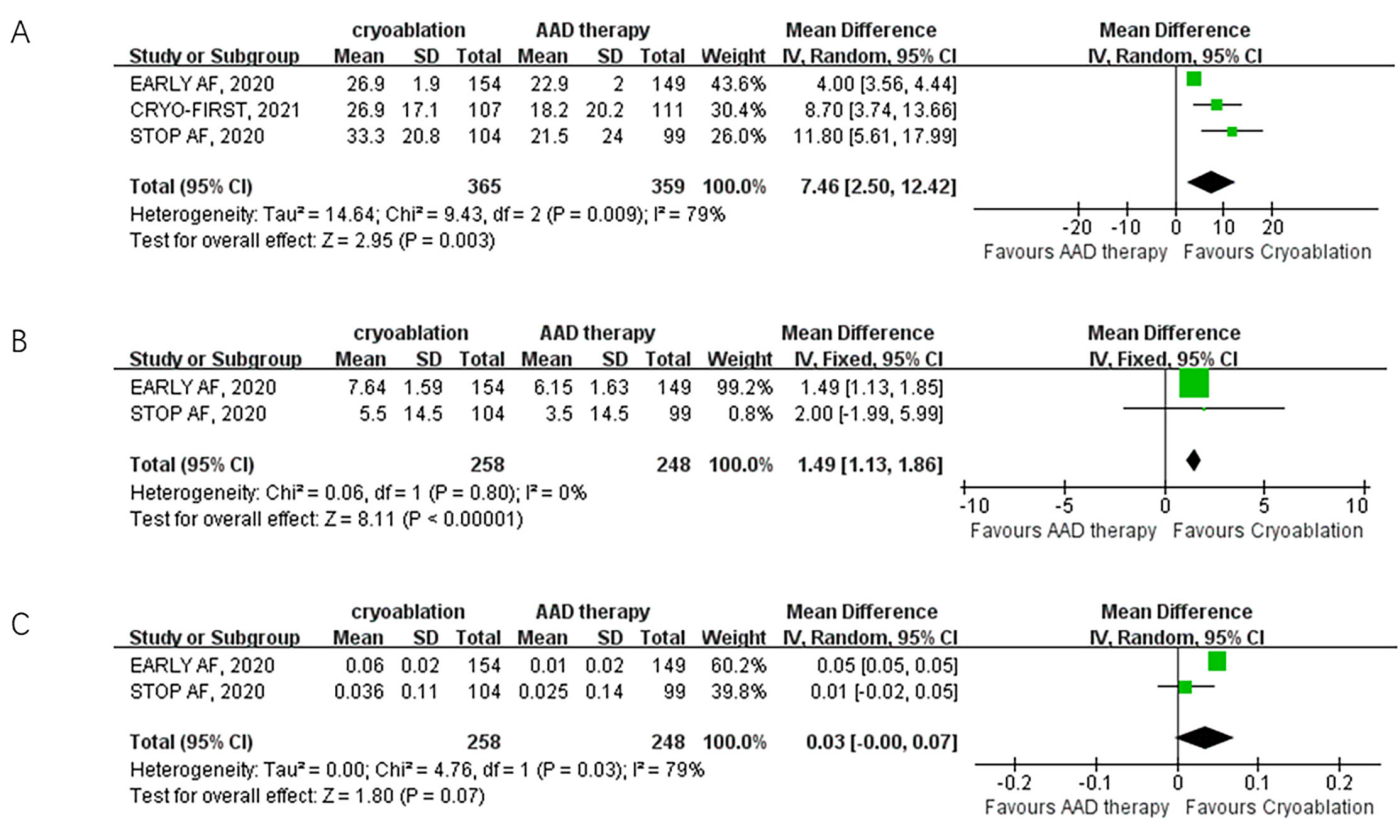
**Forest plot illustrating QoL among AF patients randomized to cryoablation versus AAD therapy**. (A) Forest plot illustrating AFEQT score among AF patients randomized to cryoablation versus AAD therapy. (B) Forest plot illustrating EQ-VAS among AF patients randomized to cryoablation versus AAD therapy. (C) Forest plot illustrating EQ-5D among AF patients randomized to cryoablation versus AAD therapy. QoL, quality of life; AFEQT, Atrial Fibrillation Effect on Quality-of-Life; EQ-VAS, European Quality of Life–visual analog scale questionnaire; EQ-5D, European Quality of Life–5 Dimensions; AF, atrial fibrillation; AAD, antiarrhythmic drug; SD, standard deviation.

**Fig. 3.**
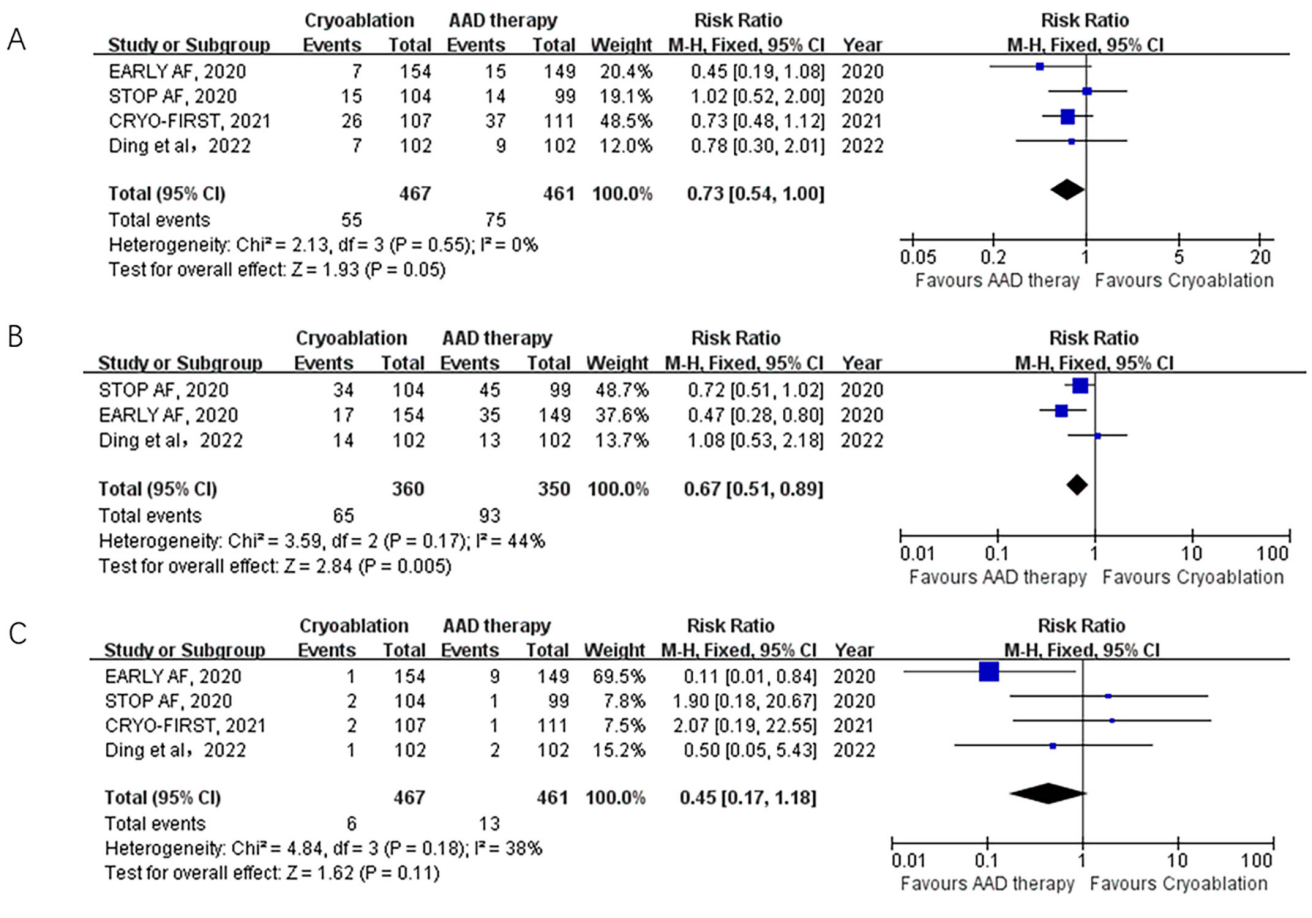
**Forest plot illustrating the safety outcomes among AF patients randomized to cryoablation versus AAD therapy**. (A) Forest plot illustrating the rate of serious adverse events among AF patients randomized to cryoablation versus AAD therapy. (B) Forest plot illustrating the rate of overall adverse events among AF patients randomized to cryoablation versus AAD therapy. (C) Forest plot illustrating the rate of MACEs among AF patients randomized to cryoablation versus AAD therapy. MCAE, major adverse cardiovascular events; AF, atrial fibrillation; AAD, antiarrhythmic drug.

The improvement in QoL, assessed using AFEQT and EQ-5D scores, was reported in the EARLY AF, CRYO-FIRST, and STOP AF trials. Three studies with a total of 724 participants reported AFEQT scores as a follow-up outcome. Cryoablation was associated with a significant increase in AFEQT scores compared to AAD therapy (3 trials; MD: 7.46, 95% CI: 2.50 to 12.42; *p* = 0.003; $I^{2}$ = 79%) (Fig. 2). Two studies with 506 participants reported EQ-5D and EQ-VAS scores as follow-up outcomes. Cryoablation demonstrated a significant improvement in EQ-VAS scores compared to AAD therapy (2 trials; MD: 1.49, 95% CI: 1.13 to 1.86; *p* < 0.001; $I^{2}$ = 0%) (Fig. 2) and no significant difference in EQ-5D scores (2 trials; MD: 0.03, 95% CI: –0.01 to 0.07; *p* = 0.07; $I^{2}$ = 79%) (Fig. 2). The primary safety outcome, serious adverse events, was reported in all 4 trials included in the analysis. The rate of serious adverse events was significantly lower with cryoablation compared to AAD therapy (4 trials; 11.8% vs. 16.3%; RR: 0.73; 95% CI: 0.54–1.00; *p* = 0.05; $I^{2}$ = 0%) (Fig. 3).

### 3.3 Secondary Outcomes

Overall adverse events were reported in 3 trials. Cryoablation was associated with a significantly lower rate of overall adverse events compared to AAD therapy (18% vs. 26.6%; RR: 0.67; 95% CI: 0.51–0.89; *p* = 0.005; $I^{2}$ = 44%) (Fig. 3). All 4 trials reported mortality outcomes, with only one death occurring in each treatment group. Major adverse cardiovascular events (MACE) percentages were lower with cryoablation compared to AAD therapy, but the difference was not statistically significant (4 trials; 1.2% vs. 2.8%; RR: 0.45; 95% CI: 0.17–1.18; *p* = 0.11; $I^{2}$ = 38%) (Fig. 3). No cases of atrioesophageal fistula, stroke, major bleeding, or symptomatic pulmonary vein stenosis were reported in the ablation group. The most common procedure-related adverse events were pericardial disorders (1.3%) and self-limited phrenic nerve injury (1.1%). The most common drug-related adverse events were bradycardia (2%) and syncope (1.3%).

Progression of AF after cryoablation or drug therapy was assessed in 2 trials involving 506 participants followed up for 36 months. The incidence of persistent atrial fibrillation was significantly lower in the ablation group compared to the drug group (2.0% vs. 11.1%; RR: 0.18; 95% CI: 0.07–0.45; *p* = 0.0002; $I^{2}$ = 0%) (**Supplementary Fig. 2**).

Hospitalizations and emergency department visits were reported in the EARLY AF and CRYO-FIRST trials. Cryoablation was associated with a 65% reduction in the rate of hospitalizations compared to AAD therapy (8.1% vs. 23.0%; RR: 0.35; 95% CI: 0.22–0.56; *p* < 0.001; $I^{2}$ = 0%) (**Supplementary Fig. 3**), which was statistically significant. Furthermore, the rate of emergency-department visits was similar in both treatment groups (19.4% vs. 25.4%; RR: 0.76; 95% CI: 0.55–1.06; *p* = 0.21; $I^{2}$ = 0%) (**Supplementary Fig. 4**).

Crossover to alternate therapy and additional ablation were reported in all 4 studies. Crossover occurred significantly less frequently in the cryoablation group compared to the AAD therapy group (1.0% vs. 10.0%; RR: 0.11; 95% CI: 0.04–0.33; *p* < 0.001; $I^{2}$ = 39%) (**Supplementary Fig. 5**). Moreover, the rate of additional ablation after initial treatment was significantly lower with cryoablation compared to AAD therapy (9.0% vs. 32.3%; RR: 0.24; 95% CI: 0.08–0.78; *p* = 0.02; $I^{2}$ = 76%) (**Supplementary Fig. 6**).

## 4. Discussion 

The principal findings of our meta-analysis provide valuable insights into the use of cryoablation as a first-line therapy for patients with symptomatic AF. We observed significant improvements in the AFEQT score with cryoablation compared to AAD therapy. However, there were no significant differences in the change in EQ-5D index score between the two treatment groups. Cryoablation also demonstrated a significant reduction in the risk of serious and overall adverse events, hospitalizations, and additional ablation compared to AAD therapy. However, there were no significant differences in major adverse cardiovascular events and emergency department visits. Importantly, we observed a lower rate of crossover to alternate therapy and additional ablation in the cryoablation group, indicating a potentially more effective and durable treatment approach.

Cryoablation has emerged as a promising approach for the treatment of AF since its introduction in 2003 [14]. This technique, characterized by a single-shot PVI, offers advantages in terms of ease of use for operators compared to the traditional “point-by-point” ablation method [27]. Numerous studies have confirmed the effectiveness of cryoablation in the treatment of AF [28]. A recent meta-analysis, which included 7195 patients from 16 studies, found similar rates of arrhythmia-free survival and adverse events between cryoablation and radiofrequency ablation, another commonly used ablation method. This suggests that cryoablation is a viable alternative with comparable outcomes to radiofrequency ablation [29]. In our analysis, all 4 trials included in the meta-analysis used second-generation cryoballoon catheters for cryoablation. These second-generation devices have demonstrated superior efficacy and a similar safety profile compared to the first-generation cryoballoon devices. This advancement in technology further supports the effectiveness and safety of cryoablation as a treatment option for AF. Overall, the evidence from our analysis and previous studies supports the validity and effectiveness of cryoablation in the treatment of AF. The use of second-generation cryoballoon (CB) catheters has further improved outcomes, making cryoablation a valuable therapeutic approach in managing cardiac arrhythmia [30].

Numerous studies have focused on comparing ablation with anti-arrhythmic drugs in patients who did not respond to drug therapy. However, recent trials have examined the use of ablation as the initial treatment for AF and compared it to drug therapy [10, 11, 12, 16, 17, 18]. In particular, one observational study demonstrated that cryoballoon-based PVI as the first-line treatment in treatment-naive patients with paroxysmal or persistent AF resulted in favorable outcomes with a 79.2% arrhythmia-free survival rate after 2 years of follow-up [31]. These findings suggest the potential of cryoablation as an initial therapy for AF in patients who have not previously received drug treatment. Previous meta-analyses comparing radiofrequency ablation with drug therapy as the initial treatment for AF showed a modest reduction in AF recurrence but no significant difference in symptomatic AF recurrence, cardiovascular outcomes, or repeated procedures [13]. Furthermore, more serious adverse events were found in the ablation arm. Recently, another meta-analysis showed a higher risk of hospitalization with ablation than with drug therapy in these three trials [32]. However, these studies reported a higher incidence of serious adverse events in the ablation group. A more recent meta-analysis, which included previous trials and three additional RCTs comparing cryoablation and drugs as the initial treatment for AF, reported improved outcomes in terms of AF recurrence and hospitalizations, with no significant difference in major adverse events [19]. It is worth noting that some information was not available for analysis due to data extraction from abstracts, and other analyses comparing cryoablation and drug therapy for initial treatment showed similar results regarding atrial arrhythmia recurrence and adverse events [20, 33].

In recent years, there has been an increasing focus on evaluating changes in symptoms and QoL with initial “first-line” therapy for paroxysmal AF [25, 26, 34]. The primary goal of AF treatment is to improve QoL and alleviate symptoms [4]. However, the clinical endpoints commonly used in cryoablation trials may not always align with the patient’s subjective perception of their overall well-being [35, 36]. Therefore, assessing QoL is crucial for a comprehensive evaluation of different treatment approaches, as it takes into account the participant’s subjective feelings about their health and considers the potential adverse effects of treatment strategies.

In our analysis, three trials evaluated QoL using various questionnaires such as the AFEQT questionnaire, the SF-36 questionnaire, and the EQ-5D and EQ-VAS questionnaires [16, 25, 26]. The AFEQT questionnaire specifically focuses on the QoL impacts of AF and is a sensitive and reliable measure for assessing disease-specific QoL [37]. We found that the AFEQT summary score significantly improved from baseline to the endpoint of follow-up in both the cryoablation and AAD groups, with a greater improvement observed in the cryoablation group. However, the results regarding general QoL tools were inconsistent. The EQ-5D and EQ-VAS questionnaires were applied in the EARLY AF and STOP AF trials, while the SF-36 questionnaire was used in the CRYO-FIRST trial. The STOP AF trial did not show significant between-group differences in the improvement of general QoL measures, which contrasts with the findings in EARLY AF and CRYO-FIRST. Despite the significant differences observed in EQ-VAS score in our analysis, it’s important to acknowledge that the results may carry limited significance, given that only two trials were included, and the analysis heavily favored one trial with a weight of 99.2%. These discrepancies may be attributed to the fact that general QoL tools do not specifically focus on the impacts of AF, potentially resulting in survey responses influenced by symptoms unrelated to the study intervention.

To assess the effect of initial rhythm control strategies on the progression of AF to a persistent form, two trials completed a 36-month follow-up [23, 24]. At 3-yearss, a significantly lower percentage of patients in the cryoablation group compared to the drug therapy group experienced progression from paroxysmal to persistent AF. Previous studies have reported that progression from paroxysmal to persistent AF occurs in 8–15% of patients at 1 year and in 22–36% of patients at 10 years after the initial onset of paroxysmal AF [23, 38, 39]. Several studies have shown that radiofrequency ablation is associated with lower rates of AF progression, and our analysis suggests that cryoablation may play a significant role in preventing the progression of AF to a persistent form [40, 41].

Safety outcomes, primarily assessed through adverse events, are a crucial consideration in the initial ablation treatment of AF. As an invasive procedure, ablation carries the potential for complications. Previous meta-analyses reported a higher incidence of serious adverse events associated with catheter ablation, although the difference was not statistically significant [13, 19]. However, our analysis suggested that cryoablation significantly reduced the rate of serious adverse events (11.8% vs. 16.3%) and overall adverse events (18% vs. 26.6%). Regarding major adverse cardiovascular events, the rates were 1.2% vs. 2.8%, which did not reach statistical significance. Importantly, there were no cases of death, atrio-esophageal fistula, stroke, major bleeding, or symptomatic pulmonary vein stenosis reported at the 12-month mark in the cryoablation group. After 12 months of follow-up, two deaths were reported in the EARLY AF trial, one of which was related to complications from acute pancreatitis in the ablation group, and the other was due to respiratory complications of amyotrophic lateral sclerosis in the drug therapy group [23]. The most common periprocedural complication observed was phrenic nerve injury, with six cases reported across the four trials, which is consistent with previous reports [31, 42]. Four cases (including one crossover case in CRYO-FIRST) of phrenic nerve injury were reversible during the follow-up period. While current guidelines recommend drug therapy as the first-line treatment for AF, it is important to note that drug therapy carries potential extracardiac and proarrhythmic side effects [43, 44]. In our analysis, 32 patients in the AAD arm discontinued AADs due to adverse effects, including withdrawal and crossover. It is worth mentioning that drug-related adverse events and procedure-related adverse events differ significantly. Additionally, the definition of major adverse events was not always consistent across the study designs, and many adverse events were uncommon or required long-term observation within the studies [45]. Thus, our analysis focused on serious adverse events, overall adverse events, and major adverse cardiovascular events. Nonetheless, a larger sample size and longer follow-up period would be necessary to thoroughly investigate the adverse event profile.

Crossover occurred in 5.3% of patients, with only three cases transitioning from cryoablation to AAD therapy. This crossover rate was lower than previously reported, primarily due to the restriction of crossover between groups in the EARLY AF trial [16]. Additionally, the rate of hospitalizations and the need for additional ablation after randomization were significantly lower in the cryoablation group compared to the drug therapy group. These findings provide further support for cryoablation as a superior first-line therapy option over pharmacological treatments in terms of safety. However, patient selection and operator experience should be taken into consideration when making treatment decisions.

## 5. Limitations

Several limitations need to be acknowledged in the present analysis. Firstly, despite all the trials included in this study being randomized controlled trials, it is important to note that neither the patients nor the physicians were blinded. The absence of blinding may have influenced the observed benefits of ablation and introduced potential bias into the results. Secondly, despite significant improvements found in AFEQT scores with cryoablation compared to AAD therapy, the heterogeneity may influence the outcome. Thirdly, the trials included in this meta-analysis were limited and the patient population in this analysis comprised younger individuals with structurally and functionally normal hearts, which could restrict the generalizability of the findings to other populations of AF patients. The outcomes and treatment effects observed in this specific patient group may not necessarily apply to older individuals or those with underlying structural heart diseases. Fourthly, continuous monitoring was only implemented in the EARLY AF trial, whereas intermittent monitoring was employed in the other studies. This disparity in monitoring methods could have led to an overestimation of the outcomes in both treatment groups. Lastly, the drug therapy group allowed the utilization of class I and III ADDs, introducing variability in drug choice and dosing. The diverse utilization of various drugs and treatment regimens among patients might have influenced the outcomes, and inadequate treatment of some patients may have contributed to an increased recurrence of atrial arrhythmias.

## 6. Conclusions

In conclusion, our meta-analysis demonstrates that cryoablation, when employed as a primary treatment modality for patients afflicted with symptomatic AF, yields substantial enhancements in AF-specific QoL and notable reductions in serious adverse events, the incidence of persistent AF, hospitalizations, and the need for additional ablation when compared to ADDs. Notably, no statistically significant disparities were discerned in general QoL, major adverse cardiovascular events or emergency department visits between the two treatment modalities. These compelling findings incontrovertibly support the adoption of cryoablation as an efficacious and secure initial therapeutic approach for symptomatic AF. Nonetheless, it is imperative to diligently contemplate the aforementioned limitations while interpreting the findings. Subsequent investigations are warranted to authenticate these findings within larger, more heterogeneous patient cohorts and to explore the enduring effects and potential benefits of cryoablation in the management of AF.

## Transparency

The lead author affirms that the manuscript is an honest, accurate, and transparent account of the study being reported; that no important aspects of the study have been omitted; and that any discrepancies from the study as planned have been explained.

## Supplementary Material

Supplementary material associated with this article can be found, in the online version, at https://doi.org/10.31083/j.rcm2502071.
