# Supplementary material for: Quality of Life and Safety Outcomes after First-Line Treatment of Symptomatic AF with Cryoablation or Drug Therapy: A Meta-Analysis of Randomized Controlled Trials
Source: Rev Cardiovasc Med. 2024 Feb 20;25(2):71. doi: 10.31083/j.rcm2502071 (PMC11263141; doi:10.31083/j.rcm2502071)
Supplement: Supplementary file 1 [file 2153-8174-25-2-071-s1.zip › 2153-8174-25-2-071-s1/Supplementary Material.doc]

**Supplementary Fig. 1. Risk of bias assessment for included studies**

**Supplementary Fig. 2.** **Forest plot illustrating the incidence of persistent atrial fibrillation among AF patients randomized to cryoablation versus AAD therapy excluding EARLY AF**. AAD, antiarrhythmic drug; AF, atrial fibrillation

**Supplementary Fig. 3. Forest plot illustrating the rate of hospitalizations among AF patients randomized to cryoablation versus AAD therapy**. AAD, antiarrhythmic drug; AF, atrial fibrillation

**Supplementary Fig. 4. Forest plot illustrating the rate of emergency department visits among AF patients randomized to cryoablation versus AAD therapy**. AAD, antiarrhythmic drug; AF, atrial fibrillation

**Supplementary Fig. 5.** **Forest plot illustrating the rate of crossovers among AF patients randomized to cryoablation versus AAD therapy**. AAD, antiarrhythmic drug; AF, atrial fibrillation

**Supplementary Fig. 6. Forest plot illustrating the rate of additional ablation among AF patients randomized to cryoablation versus AAD therapy**. AAD, antiarrhythmic drug; AF, atrial fibrillation
